# Colonization strategies of *Pseudomonas fluorescens* Pf0-1: activation of soil-specific genes important for diverse and specific environments

**DOI:** 10.1186/1471-2180-13-92

**Published:** 2013-04-27

**Authors:** Katila Varivarn, Lindsey A Champa, Mark W Silby, Eduardo A Robleto

**Affiliations:** 1 School of Life Sciences, University of Nevada Las Vegas, Las Vegas, NV, USA; 2 Department of Biology, University of Massachusetts Dartmouth, North Dartmouth, MA, USA

## Abstract

**Background:**

*Pseudomonas fluorescens* is a common inhabitant of soil and the rhizosphere environment. In addition to potential applications in biocontrol and bioremediation, *P. fluorescens* is of interest as a model for studying bacterial survival and fitness in soil. A previous study using *in vivo* expression technology (IVET) identified 22 genes in *P. fluorescens* Pf0-1 which are up-regulated during growth in Massachusetts loam soil, a subset of which are important for fitness in soil. Despite this and other information on adaptation to soil, downstream applications such as biocontrol or bioremediation in diverse soils remain underdeveloped. We undertook an IVET screen to identify Pf0-1 genes induced during growth in arid Nevada desert soil, to expand our understanding of growth in soil environments, and examine whether Pf0-1 uses general or soil type-specific mechanisms for success in soil environments.

**Results:**

Twenty six genes were identified. Consistent with previous studies, these genes cluster in metabolism, information storage/processing, regulation, and ‘hypothetical’, but there was no overlap with Pf0-1 genes induced during growth in loam soil. Mutation of both a putative glutamine synthetase gene (Pfl01_2143) and a gene predicted to specify a component of a type VI secretion system (Pfl01_5595) resulted in a decline in arid soil persistence. When examined in sterile loam soil, mutation of Pfl01_5595 had no discernible impact. In contrast, the Pfl01_2143 mutant was not impaired in persistence in sterile soil, but showed a significant reduction in competitive fitness.

**Conclusions:**

These data support the conclusion that numerous genes are specifically important for survival and fitness in natural environments, and will only be identified using *in vivo* approaches. Furthermore, we suggest that a subset of soil-induced genes is generally important in different soils, while others may contribute to success in specific types of soil. The importance of glutamine synthetase highlights a critical role for nitrogen metabolism in soil fitness. The implication of Type 6 secretion underscores the importance of microbial interactions in natural environments. Understanding the general and soil-specific genes will greatly improve the persistence of designed biocontrol and bioremediation strains within the target environment.

## Background

*Pseudomonas* spp are frequently found among the numerous bacterial genera in soil and water environments. Pseudomonads are often closely associated with animals and plants, but are also found living free in bulk soil. Apart from their probable ecological importance, several *P. fluorescens* strains are of interest as potential biological control agents. A considerable body of research has shown that secondary metabolites are critical for biocontrol, both in vitro and in greenhouse experiments [1-7]. Unfortunately, greenhouse success has not consistently translated to success in field applications. Determining mechanisms by which pseudomonads persist and compete in soil would be of use in improving biocontrol strategies as well as in deepening the understanding of microbial success within natural environments.

A substantial body of work has given insight into bacterial fitness in laboratory culture systems, and to a lesser extent genetic experiments have been used to decipher environment-specific aspects of fitness which may not be apparent during growth in laboratory media [8-11]. IVET experiments have shown that a wide range of genes are induced during growth of *Pseudomonas* sp. on plant surfaces and in soil, and these genes fall into several broad functional categories. For example, genes important for utilization of various carbon sources, basic metabolism, transport, regulation, and antisense to known genes were identified as upregulated in *Burkholderia multivorans*[8] and *P. fluorescens* Pf0-1 [11,12] during growth in soil. These studies have provided insight into genetic circuits which promote fitness, and point the way to targets which may be manipulated to improve our ability to successfully apply exogenous bacteria to soil environments. Applications which could benefit from this knowledge include biological control of plant pathogens, and bioremediation. Despite progress, our knowledge on how microbes survive and potentially adapt to new soil environments still limits further applications of the use of microbes.

Studies aimed at deciphering genetics of survival and persistence in natural environments have generally focused on the known environment of the bacterium in question. Experiments on *P. fluorescens* isolates have identified genes induced in strain SBW25 on sugar beet, the plant from which SBW25 was originally isolated, and in Pf0-1 in the soil from which it was isolated. In *P. fluorescens* Pf0-1, an antisense gene termed *cosA* was shown to be important for optimal colonization of loam soil [13] and proper regulation of the gene *ppk*, specifying polyphosphate kinase, was demonstrated to be necessary for competitive fitness [14]. In *P. fluorescens* SBW25, genes controlling production of a cellulosic polymer were implicated as important for colonization of plant surfaces [12]. While informative, these experiments do not ask what is required to colonize and persist in new environments, an ability which is critical for expanding the ecological niche of the organism and for application to new environments in biocontrol. To address this question, we used a comparative approach based on IVET technology to identify the genetic basis of adaptation of *P. fluorescens* Pf0-1 to growth in soils. This type of approach is analogous to those used in determining the least number of genes required for growth in *Staphylococcus aureus* or sporulation in the Bacilli and Clostridia [15,16]. Those studies entailed comprehensive genetic searches for factors required for growth or sporulation in a target organism and a comparative analysis to a distantly related bacterium. We examined the complement of *P. fluorescens* genes expressed in arid soil, and tested a subset of these for their effect on colonization of both arid and agricultural loam soil. Our experiments suggest that nitrogen homeostasis is a key factor in adaptation to any soil.

## Methods

### Bacterial strains, plasmids, culture conditions, and primers

Bacterial strains and plasmids used in this study are described in Table 1. Wild type *P. fluorescens* Pf0-1 and Pf0-1Δ*dap* were grown in LB [17] or *Pseudomonas* Minimal Medium (PMM) [18] at 27°C with shaking at 250 rpm. *Escherichia coli* strains were grown in LB medium at 37°C shaking at 250 rpm. Antibiotics were used at the following concentrations: ampicillin, 100 μg/ml; kanamycin, 50 μg/ml; nalidixic acid, 10 μg/ml; tetracycline, 10 μg/ml or 25 μg/ml (for *E. coli* or *P. fluorescens*, respectively); carbenicillin, 100 μg/ml; and streptomycin, 20 μg/ml. In addition, agar media used to grow Pf0-1Δ*dap,* or its derivatives, was amended with 10 μg/ml of diaminopimelic acid (DAP) (Sigma). Media used to detect β-galactosidase activity contained 35 μg/ml of X-Gal (5-bromo-4-chloro-3-indoyl-β-D-galactopyranoside). Oligonucleotide primers used in this study were synthesized by IDT (Coralville, IA) and are listed in Table 2.

**Table 1:** Bacterial strains and plasmids

**Strains, plasmids or primers**	**Genotype or description**	**Reference or source**
*E. coli*		
DH5αλ pir	φ80d*lacZ*ΔM15 Δ(*lacZYA-argF*)*U169 recA1 endA1 gyrA96 thi-1 hsdR17 supE44 relA1 deoR* λ*pir*	[19]
*P. fluorescens*		
Pf0-1	Wild type, Amp^r^	[20]
PfΔdap	Pf0-1 Δ*dapB*	This Study
Pf0-1::pKNOCK Fr2	Pf0-1 with partial deletion of *Fr2*	This Study
Pf0-1::pKNOCK Fr4	Pf0-1 with partial deletion of *Fr4*	This Study
Pf0-1::pKNOCK Fr9	Pf0-1 with partial deletion of *Fr9*	This Study
Pf0-1::pKNOCK Fr10	Pf0-1 with partial deletion of *Fr10*	This Study
Pf0-1::pKNOCK Fr2pJB	Pf0-1 with partial deletion of *Fr2* carrying pJB866	This Study
Pf0-1::pKNOCK Fr2pJB Fr2	Pf0-1 with partial deletion of *Fr2* Complemented by pJB866::Fr2	This Study
Pf0-1::pKNOCK Fr10pJB	Pf0-1 with partial deletion of *Fr10* carrying pJB866	This Study
Pf0-1::pKNOCK Fr10pJB Fr10	Pf0-1 with partial deletion of *Fr10* Complemented by pJB866::Fr10	This Study
**Plasmids**		
pGEM-T Easy	Ap^r^; cloning vector for PCR products	Promega
pIVETdap	*dapB*’ cloned in SpeI site of pUIC3	[11]
pJB866	Broad-host-range vector, Tc^r^	[21]
pRK2013	Helper plasmid, IncP Tra^+^ Mob^+^ ColE1, Km^r^	[21]
pKNOCK-Km	pBSL63 derivative carrying RP4 oriT and R6K γ-ori, Km^r^	[22]
pSR47s	Km^r^; *sacB*-containing suicide vector (requiring R6K replication origin)	[23]

**Table 2:** Oligonucleotide primer sequences

**Primers**	**Description**	**Reference or source**
Pbla	5′-CAGGGTTATTGTCTCATGAGCG-3′	[12]
Pdap	5′-CCGCCTCTACCAGCGTCTTGCC-3	[12]
DapB1	5′- GCATGAGAGCTCACCCTTTCCGTCAAAGTGC -3′	This Study
DapB2	5′- AAACCAGCGGCCGCTATACGTCGCATGCCGACTCC -3′	This Study
DapB3	5′- CGTATAGCGGCCGCTGGTTTGTACGACATGCAGG -3	This Study
DapB4	5′- TTACATGTCGACTTGCTCGCTACCAGCGG -3′	This Study
fFr2	5′- GTAACTGTTGGCCTGGAA -3′	This Study
rFr2	5′- GCCAAACGCGATCACA -3′	This Study
fFr4	5′- CCGCGTTATTCGCAGA -3′	This Study
rFr4	5′- TGTAATCATCCGGCCAGA -3′	This Study
fFr9	5′- GAGCCGACTGCACGAA -3′	This Study
rFr9	5′- TGGTCATGAGTTCGCTGA -3′	This Study
fFr10	5′- CGCACGTTCAGGCTGA -3′	This Study
rFr10	5′- CCAACAGCCACGAGCA -3′	This Study
fFr2com	5′- ATTGCGGCCGCTCAGGCTTCGGTCAGATACC-3′	This Study
rFr2com	5′- CGCACTAGTCGATGAAATTCGCAGCCATTGA -3′	This Study
fFr10com	5′- GCGCAATTCTTACTCTTTGTCCAGCATGCCA -3′	This Study
rFr10com	5′- ATTGCGGCCGCTATGAGCACTAGCGCAGCACA -3′	This Study

### DNA manipulation and sequencing

Recombinant DNA techniques were carried out as described [17] or according to supplier instructions. Restriction enzymes and DNA modifying enzymes were purchased from Invitrogen (Carlsbad, CA), New England Biolabs (Ipswich, MA), and Promega (Madison, WI). Plasmid DNA was extracted using a QIAprep Spin Miniprep kit (Qiagen, Valencia, CA). DNA fragments were recovered from agarose gel slices using a QIAquick Gel Extraction kit (Qiagen). DNA was amplified by PCR using Vent_R_ DNA polymerase (NEB). PCRs to amplify DNA for cloning were all carried out using purified genomic DNA for the template (Wizard DNA Isolation Kit, Promega). Screening of mutants was carried out by colony PCR. When required, PCR products were cloned with pGEM-T Easy (Promega). DNA sequences were determined by the Nevada Genomic Center at the University of Nevada, Reno.

### Construction of an in-frame *dapB* deletion in Pf0-1

The primer pairs DapB1/DapB2 and DapB3/DapB4 were used to PCR amplify upstream and downstream regions flanking *dapB*. The 5′ ends of DapB2 and DapB3 contained complementing linker sequences of 5′-AAACCAGCGGCCGCTATACG-3′ and 5′-CGTATAGCGGCCGCTGGTTT-3′ that were used to anneal both PCR products together. Annealed fragments were ligated into the plasmid pSR47s using the *Sal*I and *Sac*I sites, and used to transform *E. coli* DH5αλpir, resulting in pJGΔ101. The plasmid pJGΔ101 was transferred into Pf0-1 by conjugation to construct the *dapB* deletion by allele exchange, as we have described previously [11]. Deletion of *dapB* was confirmed by PCR, and by auxotrophy for DAP.

## Construction of an IVET library

A Pf0-1 genomic library was constructed in the pIVETdap vector [11]. Pf0-1 genomic DNA was extracted from a culture grown in PMM for 18 h, using the Wizard® Genomic DNA Purification Kit (Promega; Madison, WI). The genomic DNA was partially digested with four units of *Sau*3A1 (New England Biolabs, Beverly, MA) for 18 minutes. The partially digested DNA was resolved by electrophoresis, and 1 to 3 kb fragments were isolated and purified from agarose fragments using a Qiaquick gel extraction kit (Qiagen, Valencia, CA). Fragments were ligated to dephosphorylated pIVETdap (Promega Calf Intestinal Alkaline Phosphatase) linearized with *Bgl*II, yielding the pIVETdap genomic library. Library DNA was used to transform *E. coli* DH5αλpir, and clones were selected in the presence of nalidixic acid and tetracycline. A pool of 9375 clones from several independent ligations was kept at -80°C.

### Selection of soil-induced promoters

**Figure 1:**
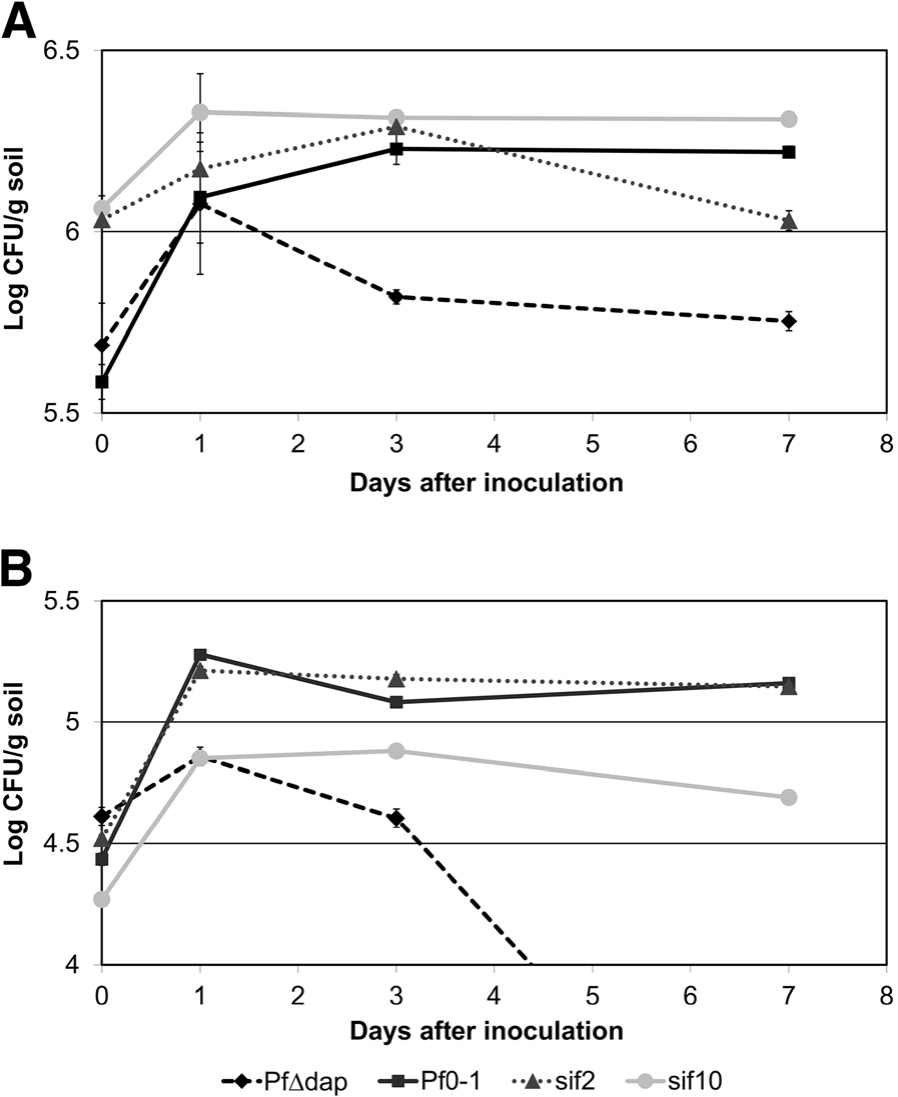
**Growth and persistence in Nevada arid soil of *P. fluorescens *Pf0-1 carrying mutations in arid soil-induced genes relative to wild-type Pf0-1 and Pf0-1Δ*dapB*. A**. When inoculated at relatively high density, the *sif2* (Pfl01_2143) mutant fails to maintain the population density reached by wild-type Pf0-1 while the *sif10* (Pfl01_5595) mutant shows no aberrant phenotype. **B**. When inoculated at relatively lower density, the *sif10* (Pfl01_5595) mutant fails to establish the same population level as wild-type Pf0-1, whereas the *sif2* (Pfl01_2143) mutant is indistinguishable from wild-type. In both panels, error bars represent 4 replications. Error bars represent standard errors. Anova for these experiments indicates significant values at P ≤0.01. For the experiments in 1A, difference values between any two means that were greater than 0.11 (day1), 0.05 (day3) and 0.08 (day7) denoted statistical significance. For the experiments in 1B, difference values between any two means that were greater than 0.07 (day1), 0.07 (day3) and 0.11 (day7) denoted statistical significance.

The Pf0-1 genomic library fused to a promoterless *dapB* in the plasmid pIVETdap (see above) was transferred by conjugation to Pf0-1Δ*dapB*. A pool of recombinant bacteria carrying pIVET fusion clones was diluted and adjusted with sterile double distilled water to 0.01 OD_550_. One mL of the bacterial suspension (approximately 5×10^5^ CFU), was used to inoculate 5 g of arid Nevada desert soil (0.91% organic matter, 89.0% sand, 4.1% silt, and 6.9% clay, with a pH level of 8.3; for a more detailed description of soil properties see [24]) in a 35 ml Pyrex test tube. Prior to inoculation Nevada soil was sifted with 1 mm^2^ screen. Inoculation resulted in a wetting event. Soil water content throughout the experiment varied from fully saturated conditions (0 kPa) to permanent wilting point (-1500 kPa). Tubes were capped. Growth and persistence in soil depends on functional DapB (Figure 1). Strains that grow in soil carry promoters in the genomic fragment which activate *dapB* transcription, thus rescuing the no-growth phenotype. To carry out two rounds of seven- day soil exposure, a soil sample of 1 g from inoculated soil was recovered, suspended in 9 mL dH_2_O, and 1mL of suspension was used to inoculate a further 5 g of soil. Bacteria were allowed to grow in this soil for an additional 7 days.

After the second 7-day period, a suspension was made from 1 g of soil (as described above), diluted, and plated onto *Pseudomonas* minimal medium supplemented with diaminopimelic acid (DAP) and X-gal, and ampicillin and tetracycline to select IVET strains. Control plates indicated that these conditions were effective at inhibiting growth of indigenous bacteria. White colonies presumed to contain soil-activated promoters fused to *dapB* were chosen for further study. We surmised that blue colonies carry fusions active in both soil and laboratory; these were not studied further.

### Sequence and promoter analysis

DNA sequences from the 30 soil induced fragments (*sif*) were blasted against the Pf0-1 annotated genome*.* Based on their match to the annotated genome, sifs were grouped into metabolism, transport, regulation and poorly characterized genes categories (Table 3). In addition to BLAST analysis, promoter scans of the regions upstream of sifs were conducted using PromScan (http://molbiol-tools.ca/promscan/), which searches for σ^54^ (σ^N^) consensus sequences [25].

**Table 3:** Arid soil-induced coding sequences

**Soil-induced fragment**	**Locus tag**	**Annotated product**	**COG ID**	**Grouping**
Nutrition and transport				
28^ab^	Pfl01_2547	Putative 4-alpha-glucanotransferase	COG1640	Carbohydrate transport and metabolism
29	Pfl01_0225	Amino acid ABC transporter, permease protein	COG0765	Amino acid transport and metabolism
2^b^	Pfl01_2143	Putative glutamine synthetase	COG1629	Amino acid transport and metabolism
Secretion				
10	Pfl01_5595	type VI secretion protein TssB2	COG3516	T6SS
Regulation				
11^a^	Pfl01_5642	Transcriptional Regulator, RpiR family	COG1737	Regulation of phosphosugarmetabolism
9^a^	Pfl01_3972	Putative diguanylate phosphodiesterase (EAL domain-containing protein)	COG2200	Signal transduction mechanisms
18	Pfl01_0719	Transcriptional Regulator, LysR family	COG0583	Transcriptional regulation
24	Pfl01_2366	Transcriptional Regulator, XRE family	COG1709	Translation, ribosomal structure and biogenesis
Defense				
4	Pfl01_2660	Putative 5-Methylcytosine-specific restriction enzyme	COG1401	Defense Mechanism
Poorly Characterized and uncharacterized				
16	Pfl01_1075	Conserved hypothetical with extensin-like domain	COG3921	Function unknown
23	Pfl01_3777	Hypothetical protein	COG0596	General function prediction only
19	Pfl01_0609	Hypothetical protein		
27^a^	Pfl01_2750	Hypothetical protein		
20	Pfl01_2901	Xylose isomerase-like TIM barrel		
Antisense^c^				
13^a^	Pfl01_3287	Putative Rho-binding antiterminator	COG4568	Transcription
8^a^	Pfl01_5547	Ribonuclease PH	COG0689	Transcription
7	Pfl01_4448	Pyruvate Kinase	COG0469	Carbohydrate transport and metabolism
12^a^	Pfl01_4455	Putative insecticidal Toxin Protein (TccC)		
25	Pfl01_4265	Cytochrome C family protein		
30^a^	Pfl01_3916	alkanesulfonate monooxygenase		
1	Pfl01_0250	TonB-dependent receptor		
21	Pfl01_2744	Putative Thiolase		
26	Pfl01_0911	Putative Fumarylacetoacetase		
3	Pfl01_5256	Putative alginate lyase		
14	Pfl01_5509	Hypothetical protein		

(a) indicates the absence of a sigma 70 promoter; (b) indicates that the region was recovered twice in independent assays; (c) for antisense loci, the annotated product refers to the coding sequence found opposite the IVET-recovered antisense sequence. Locus tag is NCBI identification number for the *P. fluorescens* coding sequences.

### Construction of mutant strains

To construct genetic variants defective in the genes expressed in arid soil conditions, internal sequences (varying from 300 to 700 bp) of *sif*2, *sif*4, *sif*9 and *sif*10 were amplified using Pf0-1 genomic DNA template and primers shown in Table 2, and cloned in pGEM®-T Easy (Promega, WI). The internal fragments of *sif*2, *sif*4, *sif*9 and *sif*10 were released from pGEM®-T Easy with *Eco*RI, and cloned into the *Eco*RI site of pKNOCK [22]. The resulting clones (pKNOCK/EcoRI: *sif*2, pKNOCK/EcoRI: *sif*4, pKNOCK/EcoRI:: *sif*9 and pKNOCK/EcoRI: *sif*10) were used to transform *E. coli* DH5αλpir, and subsequently transferred to Pf0-1 by conjugation in the presence *E. coli* carrying the helper plasmid pRK2013. Transconjugants from each mating were selected for ampicillin and kanamycin resistance, which gave rise to Pf0-1: pKNOCK *sif*2, Pf0-1: pKNOCK *sif*4, Pf0-1: pKNOCK *sif*9 and Pf0-1: pKNOCK *sif*10 respectively. These four strains were subject to the arid soil assay (described below).

### Complementation

The primer pairs fFr2com/rFr2com and fFr10com/rFr10com (Table 2) were used to amplify Pfl01_2143 (*sif2*) and Pfl01_5593 (*sif10*) from the Pf0-1 genome, respectively. Purified PCR products were digested with either *Afl*III and *Not*I (*sif2*), or *Eco*RI and *Not*I (*sif10)* and cloned into the *Afl*III/*Not*I or *Eco*RI/*Not*I sites of pJB866 respectively, yielding the complementation plasmids pJB866:: *sif2* and pJB866:: *sif10*. The complementation plasmids were transferred by conjugation into Pf0-1::pKNOCK *sif2* and Pf0-1::pKNOCK *sif10* (triparental matings with pRK2013 helper), generating Pf0-1::pKNOCK *sif*2+ *sif2* and Pf0-1::pKNOCK *sif*10+ *sif10*. The two complemented strains were subject to colonization of arid soil.

### Nevada soil growth and survival assays

Growth and survival of mutant strains in arid Nevada desert soil was carried out essentially as described in the section detailing the screening of the IVET library, with some modifications. Individual strains were grown for 20 h in PMM prior to dilution to an OD_550_ value of 0.01 or 0.001, and used to inoculate 5 g soil. Populations were monitored by periodic sampling and plating of dilutions as outlined above. The different inoculation densities were used to more fully explore colonization and persistence traits in the face of competition from indigenous microbes.

### Massachusetts soil growth and competition assays

The soil used in these experiments was a gamma irradiated fine loam from Sherborn, Massachusetts, as described [26]. Bacterial strains were grown for 16 h in PMM with appropriate antibiotics, after which cells were diluted to approximately 1×10^5^ cfu/mL in sterile distilled H_2_O (sdH_2_O). Soil growth and competition assays were carried out as described previously [14], but with the addition of 0.5% (w/w) CaCO_3_ to increase the pH to approximately 7. For soil growth experiments, 1mL of diluted cell suspension was mixed with 5 g of soil, achieving a water holding capacity of approximately 50%. For competition experiments, cultures were adjusted to equal OD_600_ values prior to dilution, and then 500 μL of each diluted competing strain were combined, and mixed with soil as for the survival experiments. Note that the OD_600_ here does not differ significantly from the OD_550_ used in the arid soil experiments. Inoculated soil samples were transferred to 15 mL polypropylene conical tubes. After 30 minutes, the initial recoverable population was established by removal of 0.5 g of soil, and recovery of and enumeration of bacteria from each sample, as we have described previously [11]. The initial populations of wild-type and mutant strains were approximately equal. The population of each member in a competing pair was monitored over time by extraction of bacteria from the soil, and determination of numbers by counting colonies on appropriate selective media [14]. The wild-type strain in competition experiments was Pf0-1Sm^r^. In wild-type vs wild-type controls, Pf0-1Sm^r^ was competed with Pf0-1Km^r^. Previous work has shown that these selective markers do not influence fitness [13,14]. The competitive index is the ratio of mutant: wild-type at a given time point divided by the initial mutant: wild-type ratio.

### Statistical tests

Statistical analyses were carried out using Microsoft Excel and GraphPad Prism v5 (GraphPad Software Inc). Specific tests are indicated in the figures in which data are presented. For the arid soil experiments, the statistical tests performed were based on ANOVAs between the strain treatments and total variance. A student′s t test with an alpha value of 0.05 was used to calculate the least significant difference between means. For competition experiments, an unpaired T-test was used, with p<0.05 used to define statistically significant differences.

## Results and discussion

### IVET selection of Pf0-1 promoters induced in arid Nevada desert soil

A library of DNA fragments, covering 94% of the *P. fluorescens* genome, was used to trap promoters induced during growth in arid Nevada desert soil, a non-native soil for Pf0-1, essentially as described previously in IVET studies of agricultural soil [11]. After two rounds of growth and enrichment in soil, bacteria which survived the soil environment were examined for expression of the fusions *in vitro* by plating onto medium containing X-gal. Thirty white colonies of the 3000 that were recovered (about 1%) contained *dapB-lacZ* fusions transcriptionally activated in soil conditions but repressed in laboratory media were chosen for further study.

The pIVETdap-based plasmids excise from the Pf0-1 genome at a low frequency, allowing recovery from the 30 strains of interest by plasmid isolation and subsequent transformation of *E. coli*. The Pf0-1 sequence fused to *dapB* in each recovered IVET plasmid was identified by DNA sequencing using the pdap primer, followed by comparison to the Pf0-1 genome sequence [27]. Sequences obtained matched predicted genes or expressed sequences antisense to predicted genes, as has been reported in previous IVET studies [for examples see [12,27-29]. Three genes, including one ‘antisense’ sequence, were recovered twice in independent selection experiments, which validated the use of IVET.

### Analysis of arid soil-activated genes

Among the 30 IVET-identified sequences isolated were representatives of several major functional groups (Table 3). Although the IVET-identified genes fell into similar broad functional categories, none of the sequences recovered here matched those results from a previous study of loam soil [11]. The lack of overlap suggests that either one or both screens provided incomplete coverage of the Pf0-1 genome, or that the transcriptional response of Pf0-1 upon introduction to arid sandy soil is distinct from that seen in loam soil. Genes we have identified as up-regulated in arid soil are predicted to have roles in metabolism, transport, and regulation. Eleven expressed sequences were reverse complements to annotated genes in the Pf0-1 genome, further supporting the suggestion that antisense regulation is widespread and important in bacteria [30-33]. Five poorly characterized/hypothetical genes were identified. The identification of novel genes induced in soil suggests that these novel functions may need to be investigated in the context of complex non-laboratory environments where their expression is induced.

We have not experimentally determined the factors in soil which induce expression of the *sif* genes, but some insight is possible from analysis of putative promoters. The antisense sequences *sif12* and *sif30* are both predicted to be preceded by sigma54-dependent promoters. In other organisms, the σ^54^-mediated response is at least in part because of nitrogen limitation, suggesting the possibility that low nitrogen levels in soil trigger expression of these antisense genes as repressors. This suggestion fits with that for *sif2* (discussed below).

#### Nutrient use and transport

Two of the soil-induced fragments (fragments 2 and 29; Table 3) are predicted to be related to amino acid production or transport. The *sif2* locus is predicted to encode a glutamine synthetase. Mutation of *sif2* does not result in glutamine auxotrophy under laboratory conditions, possibly because of the presence of the multiple genes for glutamine synthetases predicted in the Pf0-1 genome, and as has been noted previously in *Rhizobium meliloti*[34]. Amino acid transporters and a glutamine synthetase ortholog were identified in an IVET study of *Burkholderia multivorans*[8], supporting the general importance of such systems in soil, and possibly implicating nitrogen homeostasis as a critical factor for optimal growth and persistence in soil (see discussion below). Soil-induced fragment 28 is predicted to encode a 4-alpha-glucanotransferase, similar to MalQ of *E. coli*. MalQ is important in the metabolism of maltose and maltodextrins [35], possibly suggesting that maltose or maltodextrins, derived from partial hydrolysis of plant starch, are used as carbon or energy sources in arid soil.

#### Regulation

Four sequences showing similarity to members of different regulatory families were identified in our IVET screen in arid soil (Table 3). As the bacteria passage from the laboratory to the soil environment, numerous environmental parameters are altered. Many of these changes will necessitate an adaptive response by the bacterium to enable competitive survival. Up-regulation of a range of regulatory proteins has been observed previously in studies of *P. fluorescens* strains in soil and on plant surfaces [11,27] and in a study of soil-induced genes in *B*. *multivorans*[8]. While the sequence similarities of the sifs are indicative of how DNA transcription is induced, our ability to predict the nature of the environmental regulatory responses is limited by the fact that the up-regulated regulator genes do not appear to encode orthologs of functionally characterized proteins.

#### Antisense

Several IVET screens have yielded fusions to the reporter in which the annotated gene in the fusion appears to be transcribed away from the reporter [for example [8,11,29,36-38]. In the present study, 11 of 25 unique fusions were in the reverse fusion ‘antisense’ category. It has been suggested that these reverse fusions identify transcribed sequences which function as *cis*-acting antisense regulators of the annotated genes [28,29,39]. There are at least two cases showing biological relevance for *cis-acting* antisense elements in soil environments [13,40]. The reverse fusions found in this study may indicate antisense transcripts involved in controlling a range of processes: insecticidal toxin production (*sif12*); antitermination of transcription (*sif13*); pyruvate kinase (*sif7*); sulfur scavenging (*sif30*); tRNA maturation/processing (*sif8*); transport of iron or perhaps other substrates (*sif1*) [41]; degradation of alginate (*sif3*), beta oxidation of fatty acids (*sif21*), and phenylalanine or tyrosine (*sif26*). The relevance of these for colonization of soil and long term persistence remains to be explored, but it is possible to suggest a role for controlling these processes in soil. For example, it seems reasonable to speculate that cells benefit from controlling degradation of large molecules such as alginate which may have been costly to produce and could be necessary or important for survival.

Evidence for transcription of regions that produce RNA antisense to predicted genes has accumulated from genetic studies similar to this [for example [11,28,38,42], and more recently from strand-specific transcriptome sequencing [for example [43-46]. Most of these antisense RNA (asRNA) molecules are of unknown function, and are thought-provoking because they support the concept that bacterial genomes have ‘dark matter’, functional regions not easily detectable with standard gene-finding algorithms [47]. Recent functional studies have begun to assign roles to asRNA molecules [for example [13,40,44,48], and those uncovered in this study provide a rich resource for future experiments which will further expand our understanding of the genetics of soil survival and persistence.

### Soil-induced genes influence survival in arid soil

Four IVET-identified genes representing different functional classes were chosen for mutational studies. Using pKNOCK-km [22] we generated mutants of *sif2*, *4*, *9*, and *10*, and tested these for colonization of and persistence in arid soil. The mutations in *sif4* and *sif9* did not alter colonization or survival of Pf0-1 in arid soil (data not shown). In contrast, disruption of both *sif2* and *sif10* resulted in small but significant changes in the performance of Pf0-1 in arid soil.

When soil was inoculated with the *sif2* mutant diluted to above 10^5^ cfu/mL, the mutant was slightly impaired in its ability to persist over a seven day period relative to Pf0-1 (Figure 1A). The predicted role for *sif2* in nitrogen metabolism suggests that maintenance of a high population depends on the ability to assimilate sufficient nitrogen, and the *sif2* mutant is reduced in this function in soil. Under the same conditions, the *sif10* mutant showed no such defect. In contrast, when soil was inoculated with 10-fold fewer cells, the *sif10* mutant was depressed in soil colonization while the *sif2* mutant reached a similar population to the wild-type (Figure 1B). We suggest that *sif2* is important in the maintenance of high population density in soil, while the role of *sif10* is in the establishment of high density. Thus, *sif2* appears to have no effect when the inoculation is low (Figure 1B), because under these conditions Pf0-1 does not reach the density at which *sif2* is required (>6 log cfu/g of soil). Conversely, *sif10* is not necessary at higher inoculation levels (Figure 1A) because the population threshold below which *sif10* is important (<5 log cfu/g of soil) has already been surpassed. The effects of the *sif2* and *sif10* mutations were reversed by complementation (not shown).

It is important to note that the effects of *sif2* and *sif10* inactivation on soil colonization/persistence are small but significant. This was observed in independent replicate experiments that included the complemented strains (P≤0.01). The *sif2* and *sif10* regions were identified based on induction of expression and may contribute additively to arid soil colonization/persistence. The fact that one *sif*-defective strain fails to compete against the parental strain in a different environment (see section on agricultural soil) supports the notion that effects observed in arid soil were not experimental artifacts.

These two genes which were upregulated during growth in arid soil are important for optimal performance of Pf0-1 in that environment and represent attractive targets to improve persistence in bacteria applied to natural environments as biocontrol or bioremediation agents. Alternatively, identification of these sequences which contribute to fitness could add to a catalog of desirable traits which can be sought when prospecting for new biocontrol/bioremediation strains.

The *sif10* sequence identifies Pfl01_5595 as being induced in arid soil, and important for colonization of arid soil. Pfl01_5595 is predicted to be part of an HSI-II type six secretion system (T6SS) gene cluster encoded by Pfl01_5577-Pfl01_5596 [49]. T6SSs translocate effectors from the secreting cell into both eukaryote and prokaryote targets (depending on the T6SS system in question) in a contact-dependent manner reviewed in [50]. For example, *P. aeruginosa* has three T6SS gene clusters, at least two of which have distinct functions [51]. The gene Pfl01_5595 is a predicted ortholog of the *P. aeruginosa* gene PA1657 which specifies the T6SS component termed TssB, which along with TssC is thought to assemble into structures similar to bacteriophage sheath which may be contractile. This ‘sheath’ is found around a phage tail filament-like structure, and mediates the secretion of effectors into target cells [50]. T6S has been implicated in virulence toward eukaryotic hosts [for example [51-53].

Although *sif10* has not yet been experimentally confirmed to participate in T6S, we suggest that in soil *sif10* could participate in effector translocation, negatively impacting the recipient cell. In the live arid soil used here it is possible that *sif10* helps to reduce the fitness of competing bacteria by actively suppressing their growth. Many bacteria secrete antibacterial compounds into the milieu, which may inhibit competitors from a distance. However, the potential implication of T6S in fitness points toward an additional more intimate way by which bacteria may interact with and inhibit their neighbors in natural environments such as soil.

Previous studies of genes specifically induced within a given environment have yielded similar data in terms of the importance of those genes for survival or fitness. Selected environmentally induced genes from *P. fluorescens* isolates have been shown to be important in soil colonization [11] phyllosphere colonization [12], and a subset of *V. cholerae* genes induced in an infant mouse model of cholera were important for colonization [38]. The cholera study and our own unpublished data for *P. fluorescens* in agricultural soil indicate that only a subset of environmentally induced genes are necessary for full fitness in those environments, as has also been shown in the present study. It seems likely that the majority of important environmental functions have some level of functional redundancy.

### Arid soil survival genes have varied importance in agricultural soil

**Figure 2:**
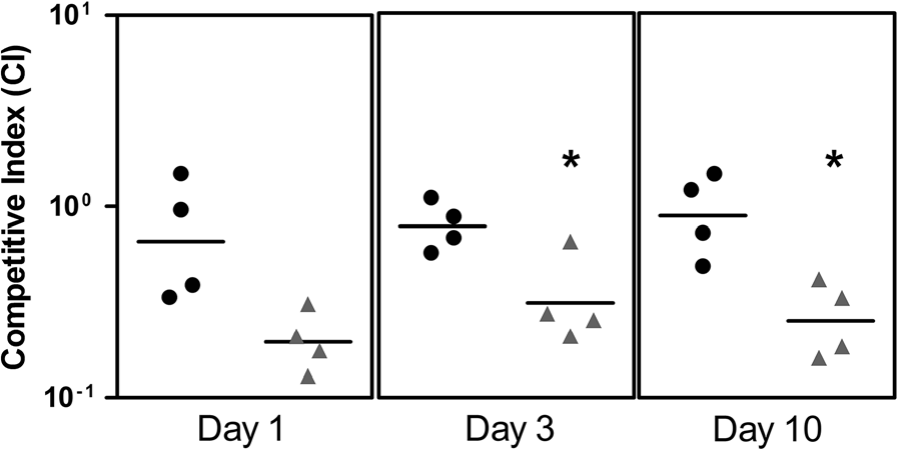
**Competitive fitness of the *sif2 *(Pfl01_2143) mutant relative to wild-type Pf0-1 in Massachusetts agricultural loam soil.** Black circle symbols represent the competitive index of the control experiment where differently marked wild-type Pf0-1 strains are competed against each other. Each data point represents the result from an independent experiment (four trials total). Neither strain has a competitive advantage. Grey triangle symbols represent the competitive index of the *sif2* mutant relative to Pf0-1. When differently marked mutant and wild-type strains are used to co-inoculate soil, the mutant is outcompeted by the wild-type. The competitive index was calculated by dividing the ratio of mutant:wildtype on a particular day by the initial ratio at the beginning of the experiment. An asterisk indicates the differences at day 3 and day 10 are significant (p<0.05; unpaired T-test).

We noted the absence of overlap between the Pf0-1 genes found to be upregulated in arid soil and those identified as upregulated in agricultural loam soil [11]. This difference could be because of limited sampling, or because of specific requirements for colonization of, and persistence in, different soil types. The soils used in these experiments differ considerably in content [24,26], and thus it might not be unexpected for different traits to be required by Pf0-1. To examine these possibilities, we tested the *sif2* and *sif10* mutants for colonization and competitive fitness in sterile agricultural loam soil as we have done in previous studies [11,14]. Neither mutant showed a colonization or persistence defect relative to Pf0-1 when inoculated alone into the sterile loam soil (not shown). However, when in competition with Pf0-1 the *sif2* mutant showed a significant competitive defect (Figure 2) while the *sif10* continued to show no discernible phenotypic difference from Pf0-1 in the agricultural soil (not shown).

The importance of *sif2* in both soil types suggests that its function in soil relates to a characteristic common to the arid and agricultural loam soils. In terms of composition, these soils are not generally similar. Physical parameters differ greatly between them, as does mineral content [24,26]. However, low inorganic nitrogen content is common between these, and probably many other soil types. The arid desert soil has a nitrate content of 15 ppm, and the agricultural loam soil used contains 69 ppm nitrate. These levels are far below those added to defined growth media used in laboratory culture such as M9 medium [17] or PMM [18]. The *sif2* sequence is predicted to specify one of several glutamine synthetases in Pf0-1. Glutamine is central to nitrogen flow in cellular metabolism, making nitrogen available for many biosynthetic reactions reviewed in [54]. Glutamine synthetases are critical players in the assimilation of nitrogen.

In *E. coli* glutamine synthetase, encoded by *glnA*, is intricately involved in nitrogen assimilation. In nitrogen-limiting conditions, expression of *glnA* is increased, thereby increasing glutamine synthetase-mediated assimilation of ammonia. Glutamine is then transformed by glutamate synthase into glutamate, which makes *glnA* the first step in ammonia assimilation. Inactivation of *glnA* renders *E. coli* auxotrophic for glutamine in conditions in which ammonia, the preferred source of inorganic nitrogen in *E. coli*, is the sole N source. Further, in N-limiting conditions the glutamine synthetase-dependent ammonia-assimilation pathway provides close to 100% of the N required in the cell. Expression of glutamine synthetase is controlled by NtrB, NtrC and GlnK, which sense glutamine levels in the cell [55].

In *Synechocystis* PCC6804, two glutamine synthetases are responsive to nitrogen availability, but differently so. The *glnN* gene is up-regulated greatly during nitrogen starvation compared to the expression level during growth in the presence of nitrate or ammonium [56].

## Conclusions

*Pseudomonas fluorescens* Pf0-1 upregulates many genes upon encountering natural environments such as soil. It is thus essential to characterize this and other bacteria in the context of their natural environments in order to gain a full appreciation of the mechanisms they use to survive and thrive. *P. fluorescens* Pf0-1 has specific genetic responses to different soil types, but also general mechanisms required for persistence. Our observation that *sif2* is important in two distinct soil types points to a general phenomenon in which bacterial responsiveness to nitrogen and its shunting into central metabolism via glutamine *in situ* is critical for fitness. This concept is further supported by the observation that several of soil-activated sequences are associated with putative σ^54^ promoters. Thus, a general key element in bacterial adaptation to soils is to maintain nitrogen homeostasis.

## Competing interests

The authors declare that they have no competing interests.

## Authors’ contribution

KV carried out the IVET screen and subsequent experiments in arid soil, and contributed to the writing of the manuscript; LC carried out experiments in agricultural soil, performed statistical tests, and contributed to manuscript writing. MS and ER designed and oversaw the study and wrote the manuscript. All authors read and approved the final manuscript.
